# Career perspectives of early-career cardiologists in the Netherlands

**DOI:** 10.1007/s12471-024-01915-2

**Published:** 2024-12-10

**Authors:** Deborah N. Kalkman, Vivan J. M. Baggen, Joost C. Beusekamp, Geert Kleinnibbelink, Wouter C. Meijers, Frederique E. C. M. Peeters, Sake J. van der Wall, Dilek Yilmaz, Madelon Minneboo, Lena Bosch

**Affiliations:** 1 Department of Cardiology, Amsterdam University Medical Centre, University of Amsterdam, Amsterdam, The Netherlands; 2 Thorax Centre, Department of Cardiology, Erasmus MC, Cardiovascular Institute, Rotterdam, The Netherlands; 3 Department of Cardiology, University Medical Center, Groningen, Groningen, The Netherlands; 4 Department of Cardiology, Radboud University Medical Centre, Nijmegen, The Netherlands; 5 Department of Cardiology, Maastricht University Medical Centre+, Maastricht, The Netherlands; 6 Department of Cardiology, OLVG Hospital, Amsterdam, The Netherlands; 7 Department of Cardiology, Leiden University Medical Centre, Leiden, The Netherlands; 8 Department of Cardiology, HMC, The Hague, The Netherlands; 9 Department of Cardiology, University Medical Centre Utrecht, Utrecht, The Netherlands

The Junior Board (De Juniorkamer) of the Netherlands Society of Cardiology (Nederlandse Vereniging voor Cardiologie) has been actively monitoring the short-term career perspectives of early-career cardiologists (‘Jonge klaren’) in the Netherlands for many years [1–4]. The objectives of these assessments are to evaluate the unemployment rate, percentage of temporary positions, job preferences and satisfaction, and demographic shifts among early-career cardiologists.

An electronic survey was distributed to all recently registered cardiologists. Hosted on the CASTOR platform, the survey comprised 42 questions covering topics such as prior training (e.g. Doctor of Philosophy (PhD) completion), type of training centre and area of expertise during training (‘Aandachtsgebied’). Additionally, career information including current position, role, fellowships and date of obtaining a permanent position was collected. Participation in the survey was voluntary and electronic informed consent was obtained.

**Fig. 1:**
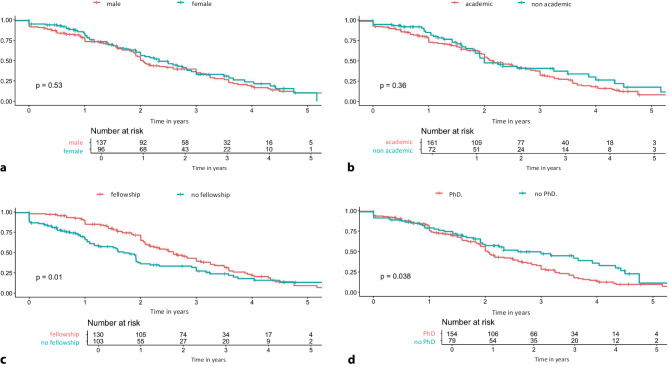
Results from the ‘Jonge klaren’ survey, percentage of temporary contracts up to 5 years after registration as a cardiologist. Subgroup analysis according to gender, academic or non-academic training centre, fellowship and PhD. **a** Percentage of temporary positions according to gender. **b** Percentage of temporary positions according to type of training centre. **c** Percentage of temporary positions according to fellowship. **d** Percentage of temporary positions according to PhD

This ‘Jonge klaren’ cohort includes early-career cardiologists who completed their training between early 2018 and early 2024. Surveys were distributed between March 2024 and June 2024, with a total of 237 responses, corresponding to a 76% response rate. Baseline characteristics showed a median age of 36 years, with 59% identified as males. Among the respondents, 69% trained at an academic medical centre, 66% had completed a PhD and 56% had conducted a fellowship. The current unemployment rate was low at 0.4% (*n* = 1), although 15% (*n* = 13) reported experiencing unemployment at some point during follow-up, with a duration ranging from 1 month to 15 months. The proportion of temporary positions at 1 year was 78%, at 2 years 53%, at 3 years 34%, at 4 years 20% and at 5 years 9%. Subgroup analyses showed no difference in the percentage of temporary positions based on gender or the type of training centre (academic vs. non-academic), but there was a significant difference in those who completed a PhD or conducted a fellowship (Fig. 1).

Overall job satisfaction was high. Using a 5-point Likert scale, with 5 being the highest level of satisfaction, over 50% of respondents rated their satisfaction as 5 and 29% rated it as 4. However, 62% of respondents reported challenges in the current job market. When asked about relocation for job opportunities, 11% were willing to move for a temporary position, while 87% would consider moving for a permanent position.

Compared with prior cohorts, there has been a decrease in the proportion of male early-career cardiologists (2011–2014 cohort: 68% male; cohort 2015–2020: 64%; cohort 2016–2021: 63%, current cohort 59%). Additionally, more respondents in the current cohort have obtained a PhD, increasing from 41% in the 2011–2014 cohort to 66% in the current cohort. A shift in training specialisation was also observed, with a decrease in interventional cardiology (16%) and increases in imaging (40%) and heart failure (22%). The proportions of respondents specialising in general cardiology (15%), electrophysiology (10%) and devices (19%) remained relatively stable. Notably, in the current cohort, no significant differences were observed between male and female respondents in terms of securing permanent positions. We consider this an important improvement compared with the first published cohort (2011–2014), in which 29% of male cardiologists obtained a permanent position at 1‑year follow-up, compared with only 12% of female cardiologists (*p* = 0.01) [1].

A significant difference in temporary position rates during follow-up remains between early-career cardiologists with and without a PhD, favouring those with a PhD. A recent survey of interns and doctors not yet in cardiology training found that 27% believed a PhD was essential for securing a cardiology training position and 52% considered it beneficial. [5] Our survey results align with this perception, showing both an earlier and higher percentage of permanent positions among PhD holders. At 3‑year follow-up, the percentage of respondents without a permanent position remained unchanged, although satisfaction with current positions showed a slight increase. Subgroup analysis by year of registration indicated a trend toward a higher percentage of permanent positions in the 2022–2024 group compared with earlier cohorts.

Limitations of this study include potential selection bias and the inherent limitations of self-reported data.

In conclusion, unemployment remains low, over one-third of respondents still hold a temporary position at 3‑year follow-up, and job satisfaction remains high. The Junior Board will continue monitoring the short-term career perspectives of ‘Jonge klaren’ in the Netherlands.
